# Parkinson's Disease and Post–COVID‐19 Syndrome: The Parkinson's Long‐COVID Spectrum

**DOI:** 10.1002/mds.28622

**Published:** 2021-04-28

**Authors:** Valentina Leta, Mayela Rodríguez‐Violante, Arturo Abundes, Katarina Rukavina, James T. Teo, Cristian Falup‐Pecurariu, Laura Irincu, Silvia Rota, Roongroj Bhidayasiri, Alexander Storch, Per Odin, Angelo Antonini, Kallol Ray Chaudhuri

**Affiliations:** ^1^ King's College London, Department of Neurosciences Institute of Psychiatry, Psychology & Neuroscience London UK; ^2^ Parkinson's Foundation Centre of Excellence King's College Hospital London UK; ^3^ Clinical Neurodegenerative Research Unit National Institute of Neurology and Neurosurgery Mexico City Mexico; ^4^ Movement Disorder Clinic National Institute of Neurology and Neurosurgery Mexico City Mexico; ^5^ King's College Hospital National Health Service Foundation Trust London UK; ^6^ Department of Neurology Faculty of Medicine, Transilvania University of Brașov Brașov Romania; ^7^ Department of Neurology County Emergency Clinic Hospital Brașov Romania; ^8^ Chulalongkorn Centre of Excellence for Parkinson's Disease & Related Disorders, Department of Medicine Faculty of Medicine, Chulalongkorn University and King Chulalongkorn Memorial Hospital Bangkok Thailand; ^9^ The Academy of Science The Royal Society of Thailand Bangkok Thailand; ^10^ German Center for Neurodegenerative Diseases Rostock/Greifswald Rostock Germany; ^11^ Department of Neurology Translational Neurodegeneration Section “Albrecht Kossel,” University of Rostock Rostock Germany; ^12^ Division of Neurology, Department of Clinical Sciences Lund Lund University Lund Sweden; ^13^ Parkinson and Movement Disorders Unit, Department of Neuroscience University of Padua Padua Italy

**Keywords:** COVID‐19, Parkinson's disease, SARS‐CoV‐2, Long COVID, Post–COVID‐19 syndrome

Implications of severe acute respiratory syndrome coronavirus 2 (SARS‐CoV‐2) infection in Parkinson's disease (PD), particularly worsening of motor and non‐motor symptoms and possibly higher mortality in those with advanced disease, comorbidities, and frailty have been reported in several case series and observational studies.^1^, ^2^, ^3^, ^4^, ^5^ As time has evolved, the issue of long‐term sequelae in patients affected by coronavirus disease 2019 (COVID‐19), often referred to as “long COVID”, has emerged, and recently, in the United Kingdom, the National Institute for Health and Care Excellence has defined the “post–COVID‐19 syndrome” as “signs and symptoms that develop during or after an infection consistent with COVID‐19, continue for more than 12 weeks and are not explained by an alternative diagnosis.”^6^ Here we present the prevalence of post–COVID‐19 syndrome in 27 patients with PD who were affected by COVID‐19 across several centers in the United Kingdom, Italy, Romania, and Mexico from the beginning of March 2020 to present (Table 1). As some of the post–COVID‐19 symptoms may be part of the PD clinical phenomenology, we considered symptoms part of the clinical manifestations of a post–COVID‐19 syndrome only if these occurred after a confirmed SARS‐CoV‐2 infection or in case of an acute or subacute worsening of a preexisting symptom that had been previously stable. In addition, we report on motor worsening and increased levodopa equivalent daily dose requirements within the long‐COVID spectrum. In our series, 23 (85.2%) patients with PD developed post–COVID 19 symptoms (Table 1). We report that the most common long‐term effects of COVID‐19 are worsening of motor function (51.9%) and increased levodopa daily dose requirements (48.2%) followed by fatigue (40.7%); cognitive disturbances (22.2%), including “brain fog”, loss of concentration and memory deficits; and sleep disturbances (22.2%), such as insomnia. Broadly these symptom complexes concur with the existing literature on long COVID in the general population.^7^ Interestingly, the severity of COVID‐19, as indicated by a history of hospitalization, did not seem to be the *condicio sine qua non* for the development of a post–COVID‐19 syndrome in patients with PD. We also believe that in some cases the stress of a prolonged lockdown due to the pandemic and the reduced access to health care and rehabilitation interventions may contribute to the burden of the post–COVID‐19 syndrome in PD. Therefore, post‐COVID clinical manifestations may result from a combination of new symptoms and lockdown as well as viral illness‐related worsening of preexisting PD features.

**TABLE 1:** Demographics, PD‐related information, and prevalence of post–COVID‐19 syndrome (n = 27); Data is presented as mean ± standard deviation, median (interquartile range) or number (percentage)

Outcome measures	Results
Sex, male	16 (59.3%)
Race/ethnicity	
White	9 (33.3%)
Black	2 (7.4%)
Other	16 (59.3%)
Age at PD diagnosis, years	59.0 ± 12.7
PD disease duration at time of COVID‐19 diagnosis, years	9.2 ± 7.8
H&Y stage at COVID‐19 diagnosis	2.0 (1.0)
LEDD at COVID‐19 diagnosis, mg	1053.5 ± 842.4
Hospitalization due to COVID‐19	6 (22.2%)
Charlson Comorbidity Index at COVID‐19 diagnosis	2.0 (1.5)
Post–COVID‐19 syndrome	23 (85.2%)
Respiratory symptoms	
Breathlessness	1 (3.7%)
Cough	3 (11.1%)
Cardiovascular symptoms	
Chest tightness	0 (0%)
Chest pain	1 (3.7%)
Palpitations	0 (0%)
Generalized symptoms	
Fatigue	11 (40.7%)
Fever	5 (18.5%)
Pain	3 (11.1%)
Neurological symptoms	
Cognitive disturbances^a^	6 (22.2%)
Headache	5 (18.5%)
Sleep disturbances	6 (22.2%)
Peripheral neuropathy symptoms^b^	3 (11.1%)
Dizziness	4 (14.8%)
Delirium	2 (7.4%)
Gastrointestinal symptoms	
Abdominal pain	0 (0%)
Nausea	2 (7.4%)
Diarrhea	0 (0%)
Reduced appetite	1 (3.7%)
Musculoskeletal symptoms	
Joint pain	3 (11.1%)
Muscle pain	2 (7.4%)
Psychological/psychiatric symptoms	
Depression	2 (7.4%)
Anxiety	4 (14.8%)
Ear, nose, and throat symptoms	
Tinnitus	0 (0%)
Earache	0 (0%)
Sore throat	0 (0%)
Loss of taste or smell	4 (14.8%)
Dermatological	
Skin rashes	0 (0%)
PD‐specific aspects	
Motor worsening	14 (51.9%)
Increased LEDD requirement	13 (48.2%)

^a^ Brain fog, loss of concentration, or memory issues.

^b^ Pins and needles and numbness.

Abbreviations: PD, Parkinson's disease; COVID‐19, coronavirus disease 2019; SD, standard deviation; IQR, interquartile range; H&Y, Hoehn and Yahr Scale; LEDD, levodopa equivalent daily dose.

In conclusion, this is the first multicenter case series investigating the occurrence of post–COVID‐19 syndrome in patients with PD. The small sample size and the lack of a controlled group make it challenging to draw any firm conclusions; nevertheless, we believe our case series is meaningful as it highlights the possibility of the existence of a post–COVID‐19 syndrome in most patients with PD recovering from acute COVID‐19. There is a clear need for greater awareness of this issue among health care professionals, and further studies are required with longitudinal follow‐up of a larger cohort of patients with PD to address the natural history of the reported symptoms and with a view to developing personalized management strategies.

## Author Roles

(1) Research Project: A. Conception, B. Organization, C. Execution; (2) Statistical Analysis: A. Design, B. Execution, C. Review and Critique; (3) Manuscript: A. Writing of the First Draft, B. Review and Critique.

V.L.: 1A, 1B, 1C, 2A, 2B, 2C, 3A, 3B

M.R.V.: 1C, 3B

A. Abundes: 1C, 3B

K.R.: 1C, 3B

J.T.T.: 1C, 3B

C.F.P.: 1C, 3B

L.I.: 1C, 3B

S.R.: 1C, 3B

R.B.: 1C, 3B

A.S.: 1C, 3B

P.O.: 1C, 3B

A. Antonini: 1C, 3B

K.R.C.: 1A, 1B, 2C, 3B

## Financial Disclosures of All Authors (for the Preceding 12 Months)

V.L. received honoraria for speaker‐related activities from Bial, Profile, and Britannia Pharmaceuticals. J.T.T. received research support and funding from Office of Life Sciences, InnovateUK, Bristol‐Myers‐Squibb, iRhythm Technologies, and received speaker honorarium from Bristol‐Meyers‐Squibb and Goldman Sachs. P.O. received compensation for lectures and advice from AbbVie, Bial, Britannia, Ever Pharma, Global Kinetics (GKC), Lobsor, Nordic Infucare, and Zambon. R.B. receives salary from Chulalongkorn University and stipend from the Royal Society of Thailand; has received consultancy and/or honoraria/lecture fees from Abbott, Boehringer‐Ingelheim, Britannia, Ipsen, Novartis, Teva‐Lundbeck, Takeda, and Otsuka pharmaceuticals; has received research funding from the Newton Fund, the UK government, Thailand Science, and Research Innovation Bureau, Thailand Research Fund, Crown Property Bureau, Chulalongkorn University, and the National Science and Technology Development Agency; and holds patents for a laser‐guided walking stick, portable tremor device, nocturnal monitoring, and electronic Parkinson's disease symptom diary as well as copyright on Parkinson's mascot, dopamine lyrics and teaching video clips for common nocturnal and gastrointestinal symptoms for Parkinson's disease. A. Antonini has received compensation for consultancy and speaker related activities from Union Chimique Belge (UCB), Boehringer Ingelheim, Britannia, AbbVie, Kyowa Kirin, Zambon, Bial, Neuroderm, Theravance Biopharma, Roche and receives research support from Chiesi Pharmaceuticals, Bial, Lundbeck, Horizon 2020 (Grant 825,785), Horizon2020 (Grant 101,016,902), Ministry of Education University and Research (Grant ARS01_01081), and the Cariparo Foundation. He serves as consultant for Boehringer–Ingelheim for legal cases on pathological gambling. K.R.C. has received honoraria for advisory boards from AbbVie, UCB, Pfizer, Jazz Pharma, GKC, Bial, Cynapsus, Novartis, Lobsor, Stada, Medtronic, Zambon, Profile, Sunovion, Roche, Theravance, and Scion; honoraria for lectures from AbbVie, Britannia Pharmaceuticals, UCB, Mundipharma, Zambon, Novartis, Boehringer Ingelheim, Neuroderm, and Sunovion; grants (investigator initiated) from Britannia Pharmaceuticals, AbbVie, UCB, GKC, and Bial; and academic grants from Innovative Medicines Initiative European Union (IMI EU), Parkinson's UK, National Institute for Health Research, Parkinson's disease non‐motor study group (PDNMSG), European Union (EU) (Horizon 2020), Kirby Laing Foundation, Parkinson's Foundation (PF) (USA), and Medical Research Council (MRC).
